# Progression patterns under BRAF inhibitor treatment and treatment beyond progression in patients with metastatic melanoma

**DOI:** 10.1002/cam4.1267

**Published:** 2017-12-20

**Authors:** Jessica C. Hassel, Kristina Buder‐Bakhaya, Carolin Bender, Lisa Zimmer, Benjamin Weide, Carmen Loquai, Selma Ugurel, Alla Slynko, Ralf Gutzmer, the German Dermatooncology Group (DeCOG/ADO)

**Affiliations:** ^1^ Department of Dermatology and National Center for Tumor Diseases University Hospital Heidelberg Heidelberg Germany; ^2^ Department of Dermatology University Hospital Essen University Duisburg‐Essen Essen Germany; ^3^ Department of Dermatology Center for Dermatooncology University Medical Center Tübingen Tübingen Germany; ^4^ Department of Dermatology University Medical Center Johannes Gutenberg‐University Mainz Germany; ^5^ Department of Mathematics, Natural and Economic Sciences University of Applied Sciences Ulm Germany; ^6^ Department of Dermatology and Allergy Skin Cancer Center Hannover Hannover Medical School Hannover Germany

**Keywords:** BRAF inhibitor, BRAF mutation, dabrafenib, metastatic melanoma, progression, treatment beyond progression, vemurafenib

## Abstract

Despite markedly improved treatment options for metastatic melanoma, resistance to targeted therapies such as BRAF inhibitors (BRAFi) or BRAFi plus MEK inhibitors (MEKi) remains a major problem. Our aim was to characterize progression on BRAFi therapy and outcome of subsequent treatment. One hundred and eighty patients with BRAF‐mutant metastatic melanoma who had progressed on treatment with single‐agent BRAFi from February 2010 to April 2015 were included in a retrospective data analysis focused on patterns of progression, treatment beyond progression (TBP) and subsequent treatments after BRAFi therapy. Analysis revealed that 51.1% of patients progressed with both new and existing metastases opposed to progression of only preexisting (28.3%) or only new (20.6%) metastases. Exclusive extracranial progression occurred in 50.6% of patients compared to both extra‐ and intracranial (29.4%) or sole cerebral progression (20%). Multivariable analyses demonstrated that single site progression and primary response to BRAFi were associated with improved progression‐free survival. Progression with exclusively new or only existing metastases and a baseline Eastern Cooperative Oncology Group (ECOG) of 0 were associated with prolonged overall survival (OS). TBP had no significant impact on OS. Other subsequent treatments showed low efficacy with the exception of anti‐PD‐1 antibodies. In conclusion we identified specific patterns of progression which significantly correlate with further prognosis after progression on BRAFi treatment. In contrast to previously published data, we could not demonstrate a significant survival benefit for BRAFi TBP. Subsequent therapies had strikingly low efficacy except for PD‐1 inhibitors.

## Introduction

BRAF inhibition (BRAFi) and combined BRAF/MEK inhibition (BRAFi/MEKi) have shown significant clinical activity in patients with BRAF V600‐mutant advanced melanoma with response rates between 45% and 53% (monotherapy) and 64% and 68% (combination treatment) 1, 2, 3, 4, 5. Although the addition of the MEKi prolonged progression‐free survival (PFS) and overall survival (OS) compared to BRAFi monotherapy, emergence of resistance remains the major clinical problem 6. Mechanisms of resistance include the reactivation of the MAPK pathway, for example, by mutations in NRAS or MEK as well as mutations leading to the activation of other proliferative signaling pathways such as PIK3CA and PTEN 7. Interestingly, PFS and OS are markedly shorter in patients with a high tumor load measured by the serum lactate dehydrogenase (LDH), and metastases in more than two disease sites 8, 9. Patients who develop long‐term responses typically show limited disease at treatment start 8, 9.

The aim of this study was to assess the patterns of progression on BRAFi and to evaluate clinical characteristics predictive of survival and response to BRAFi in a large cohort of melanoma patients. Furthermore, we addressed the question of efficacy of treatment after progression on BRAFi treatment.

## Materials and Methods

### Patients

One hundred and eighty treatment‐naïve or pretreated patients with BRAF‐mutant, metastatic melanoma of unresectable stage III or IV (American Joint Committee on Cancer [AJCC; 10]) who had progressed on treatment with single‐agent dabrafenib (Novartis, Nürnberg, Germany) or vemurafenib (Roche, Germany, Grenzach‐Wyhlen) from February 2010 to April 2015 were included in our multicenter retrospective data analysis. These consisted of patients with primary BRAFi resistance as well as patients who first responded to treatment and developed secondary resistance. The study was approved by the Ethics Committee, Hannover Medical School, Hannover, Germany.

### Data collection

Data were collected in a standardized way using a questionnaire assessing demographics and clinical characteristics at the start of BRAFi therapy and at time of disease progression including patients sex, age, prior treatment, BRAF mutational status, at treatment start and stop date of BRAFi treatment, LDH and S‐100 at start and at progression, Eastern Cooperative Oncology Group (ECOG) at treatment start start and progression, best response, date of best response, date of progression, patterns of progression I, II, and III (see below), further treatment after progression on BRAFi, response to subsequent treatments, date of death or last contact, respectively. Radiology reports were reviewed locally to determine the pattern of disease progression focused on (1) discrimination by development of new lesions only, progression of existing metastases only or both new and progression of existing metastases (*pattern I*); (2) discrimination by progression in the central nervous system (CNS) only, non‐CNS progression only, and both CNS‐ and non‐CNS progression (*pattern II*); (3) discrimination by progression of metastases that had completely disappeared at a prior staging, and presence of controlled metastases despite progression of other lesions (p*attern III*).

### Statistical analysis

Response to treatment was evaluated by Response Evaluation Criteria in Solid Tumors (RECIST) version 1.1 criteria 11. Response to therapy was defined either as objective response (complete remission [CR] or partial response [PR]) versus nonresponse (progression [PD] or stable disease [SD] or via disease control [DC], i.e., CR, PR, or SD) versus PD patients. Univariable and multivariable response analyses were performed using logistic regression. Group comparisons were carried out by Fisher exact test or Mann–Whitney test whenever suitable. *P*‐values were considered statistically significant with *P *<* *0.05. All regression analyses were performed as complete case analyses, with patients with missing observations being excluded from the respective analysis. The number of patients considered in each analysis is provided in the corresponding table. Baseline PFS and OS I were measured as the time from BRAFi commencement to the time of disease progression and date of death, respectively. OS II was calculated from the progression date under BRAFi treatment to date of death (landmark method) 12. Living patients were censored at the last contact date. All factors assumed being clinically relevant for OS were analyzed by means of the Cox proportional hazard (PH) regression model, both in a univariable and multivariable setting. A number of prognostic factors with a presumable impact on survival were measured at progression date at earliest; thus the starting points of the respective analyses were shifted to this date. Since no survival analysis techniques could be applied to PFS in such cases, logistic regression was used. Logistic regression was also applied for OS analyses in case the assumptions of the Cox PH model were not satisfied. The statistical analyses were performed using R version 3.4.0 (The R Foundation for Statistical Computing, Vienna, Austria).

## Results

### Patient characteristics

Of 180 included patients, the majority was treated with vemurafenib (82.4%; Table S1). Most of the melanomas (68.3%) carried a BRAF V600E mutation. At start of BRAFi LDH levels were elevated in half of the patients. Ninety‐seven of 180 patients (53.9%) were pretreated, namely, with dacarbazine (*n *=* *52), polychemotherapy (*n *=* *11), ipilimumab (*n *=* *19), vaccines (*n *=* *24), or others (Table S1). 51.7% of patients showed an objective response to BRAFi treatment, SD was seen in 25.0%, whereas 23.3% of patients were primary resistant to the BRAFi treatment (Table S1). Median PFS was 4.6 months (95% CI 3.9–5.5 months) and median OS was 9.8 months (95% CI 9.1–11.7 months). Until data cut‐off 165 patients (91.7%) had died; median follow‐up time in living patients was 3.2 years (range 0.3–5.6 years).

### Pattern of BRAFi disease progression

**Figure 1:**
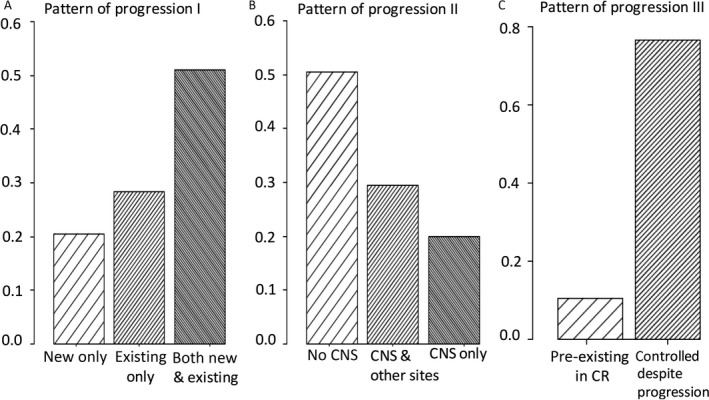
(A) Patterns of progression I includes discrimination by development of new lesions only (20.6%), progression of existing metastases only (28.3%) or both new and progression of existing metastases (51.1%). (B) Pattern of progression II discriminates by progression in the central nervous system (CNS) only (20.0%), non‐CNS progression only (50.6%), and both CNS‐ and non‐CNS progression (29.4%). (C) Pattern III includes discrimination by progression of metastases that had completely disappeared at a prior staging (10.6%), and presence of controlled metastases despite progression of other lesions (76.7%).

Concerning the general pattern of progression (*pattern I*), more than half of the patients (51.1%) progressed with both new and existing metastases (Fig. 1A; Table S2). Progression with only preexisting or new metastases was seen in 28.3% and 20.6% of patients, respectively. The majority of patients progressed with involvement of non‐CNS sites (50.6%) or both CNS and non‐CNS sites (29.4%), whereas 20.0% of patients showed sole cerebral progression (*pattern II*, Fig. 1B). Progression of sole metastases that were initially in CR during BRAFi therapy was seen in 10.6% of patients, and controlled metastases despite progressing lesions were present in 76.7% of patients (Fig. 1C, *pattern III*). Most frequent sites of progression were lymph nodes, lung, CNS, and liver. 18.3% of patients progressed in a single site or organ (further termed “single site”; Table S2).

### Prognostic factors at start of BRAFi treatment for response, duration of response, and survival

The response groups were well balanced concerning age, sex, the number previous therapies, and the type of BRAFi (Table S3). Primary response was significantly associated with normal baseline serum LDH and the BRAF V600 mutation status in univariable analysis (Table S3). Regarding the site of progression, patients who primarily showed response were less likely to progress with liver, lung and liver, or CNS metastases (Table S3). In a multivariable model that controls for baseline LDH, baseline ECOG, and BRAF mutation type only baseline LDH remained a statistically significant factor (OR=0.20, 95% CI: 0.08–0.49; *P *<* *0.001, Table 1). Data concerning DC are given in Table S3.

**Table 1:** Multivariable response‐to‐therapy analyses (logistic regression, *N *=* *102)

Risk factor	Response versus non‐response groups		Disease control versus progression groups	
	OR (95% CI)	*P*‐value	OR (95% CI)	*P*‐value
Elevated LDH at baseline				
Yes	**0.20 (0.08; 0.49)**	**<0.001**	**0.32 (0.09; 0.93)**	**0.045**
No				
BRAF mutation				
V600E				
V600K	0.17 (0.01; 1.17)	0.121	0.39 (0.07; 2.42)	0.293
Unknown	0.91 (0.35; 2.44)	0.856	0.78 (0.26; 2.49)	0.671
ECOG‐PS at baseline				
0				
≥1	1.10 (0.45; 2.79)	0.833	**0.22 (0.06; 0.68)**	**0.014**

ECOG‐PS, Eastern Cooperative Oncology Group Performance Score; LDH, lactate dehydrogenase; *N*, number of patients available for analysis; OR, odds ratio.

Univariable analyses comparing patients below or above the median PFS revealed normal baseline LDH and S‐100, baseline ECOG of 0, response to BRAFi therapy, progression in a single site only and progression with only new metastases to be significantly associated with improved PFS (Table S4). In a multivariable analysis (*n *=* *162), best response to BRAFi therapy (OR=0.26, 95% CI: 0.11–0.6; *P *<* *0.01) and single site progression (OR=3.23, 95% CI: 1.17–10.05; *P *=* *0.03) remained significant (Table 2).

**Table 2:** Multivariable analyses for PFS (logistic regression, *N *=* *162)

Risk factor	OR (95% CI)	*P*‐value
Elevated LDH at baseline		
Yes	0.65 (0.30; 1.43)	0.284
No		
Best response		
CR and PR		
SD	**0.26 (0.11; 0.6)**	**<0.01**
PD	**0.03 (0.01; 0.11)**	**<0.001**
Single site progression		
Yes	**3.23 (1.17; 10.05)**	**0.030**
No		

CR, complete response; *N*, number of patients available for analysis, OR, odds ratio; PD, progressive disease; PR, partial response; SD, stable disease; PFS, progression‐free survival; LDH, lactate dehydrogenase.

Baseline ECOG of ≥1, progression with both new *and* existing metastases, progression of both extra‐ *and* intracranial metastases, and progression with bone metastases were significantly associated with a shorter OS in a univariable analysis (Table S5). Elevated baseline LDH (OR=0.22, 95% CI: 0.11–0.43; *P *<* *0.001) and S‐100 (OR=0.098, 95% CI: 0.02‐0.31; *P *<* *0.001) showed a significant association with a shorter OS (logistic regression, groups divided based on median OS). In a multivariable model while controlling for pattern of progression I and II, single site progression, subsequent treatment and baseline ECOG (*n *=* *103), progression with new *and* existing metastases (HR=3.61, 95% CI: 1.79–7.25; *P *<* *0.001), and baseline ECOG ≥1 (HR=1.87, 95% CI: 1.2–2.89; *P *=* *0.005) were factors for shorter OS (Table 3).

**Table 3:** Multivariable analyses for OS (Cox PH model, *N *=* *103)

Risk factor	Median OS (95% CI), in months	HR (95% CI)	*P*‐value
ECOG‐PS at baseline			
0	15.13 (11.6; 20.5)		
≥1	8.27 (7.0; 9.83)	**1.87 (1.2; 2.89)**	**0.005**
Pattern of progression I			
New only	7.58 (5.80; 12.58)		
Existing only	6.29 (4.27; 10.52)	1.85 (0.94; 3.64)	0.075
Both new and existing	3.10 (2.53; 4.14)	**3.61 (1.79; 7.25)**	**<0.001**
Pattern of progression II			
No CNS	5.66 (4.37; 7.78)		
CNS and other sites	3.29 (2.00; 4.90)	1.38 (0.84; 2.27)	0.198
CNS only	5.75 (3.32; 7.48)	1.91 (0.99; 3.66)	0.052
Pattern of progression III			
Preexisting in complete remission			
Yes	6.29 (4.18; 24.5)	0.53 (0.28; 1.0)	0.052
No	4.90 (3.52; 5.80)		
Single site progression			
Yes	10.52 (6.9; 17.26)	0.76 (0.41; 1.41)	0.382
No	3.78 (3.18; 4.97)		
Subsequent treatment			
Yes	6.71 (5.80; 8.76)	**0.35 (0.22; 0.55)**	**<0.001**
No	2.00 (1.35; 3.10)		

BRAFi, BRAF inhibitor; ECOG‐PS, Eastern Cooperative Oncology Group Performance Status; HR, hazard ratio; *N*, number of patients available for analysis; OS, overall survival; CNS, central nervous system; PH, proportional hazard.

### Further treatments after disease progression on the BRAFi

In 47 of 180 patients (26.1%) the BRAFi was continued beyond disease progression. These were more frequently patients with better prognostic features like only new metastases (*P *=* *0.015) and a lower LDH (*P *=* *0.076; Table 4). Additional treatments to treatment beyond progression (TBP) included surgery (*n *=* *11) and/or radiation therapy (*n *=* *17). Two patients received additional ipilimumab (*n *=* *2), and 18 of 47 (38.3%) patients received no additional treatment.

**Table 4:** Comparison of patient groups with BRAFi treatment versus without BRAFi treatment beyond progression

Risk factor	*N*	Patients with TBP *n *=* *47 (26.1%)	Patients without TBP *n *=* *133 (73.9%)	*P*‐value
Median age at progression, (range), in years	180	56.31 (24.39; 99.53)	51.32 (19.45; 86.33)	0.226
BRAF mutation	180			
V600E		31 (66.0%)	92 (69.2%)	0.066
V600K		0 (0.00%)	10 (7.50%)	
Unknown		16 (34.0%)	31 (23.3%)	
Previous treatment (before BRAFi)	179			
Yes		29 (61.7%)	68 (51.1%)	0.164
No		17 (36.2%)	65 (48.9%)	
Elevated LDH at progression	138			
Yes		12 (25.5%)	56 (42.1%)	0.076
No		22 (46.8%)	48 (36.1%)	
Unknown		13 (27.7%)	29 (21.8%)	
Elevated S100 at progression	66			
Yes		11 (23.4%)	31 (23.3%)	0.114
No		11 (23.4%)	13 (9.80%)	
Unknown		25 (53.2%)	89 (66.9%)	
ECOG at progression	99			
0		9 (19.10%)	21 (15.8%)	0.623
≥1		17 (36.2%)	52 (39.1%)	
Unknown		21 (44.7%)	60 (45.1%)	
Pattern of progression I	180			
New only		15 (31.9%)	22 (16.5%)	**0.015**
Existing only		16 (34.0%)	35 (26.3%)	
Both new and existing		16 (34.0%)	76 (57.1%)	
Pattern of progression II	180			
No CNS		21 (44.7%)	70 (52.6%)	0.454
CNS and other sites		14 (29.8%)	39 (29.3%)	
CNS only		12 (25.5%)	24 (18.1%)	
Pattern of progression III				
Preexisting in CR	176			
Yes		7 (14.9%)	12 (9.0%)	0.275
No		39 (83.0%)	118 (88.7%)	
Controlled despite progression	177			
Yes		36 (76.6%)	102 (76.7%)	0.995
No		10 (21.3%)	9 (21.8%)	
Single site progression	180			
Yes		6 (12.80%)	27 (20.3%)	0.282
No		41 (87.2%)	106 (79.7%)	
Sites of progression				
Lymph nodes metastases	180			
Yes		30 (63.8%)	77 (57.9%)	0.495
No		17 (36.2%)	56 (42.1%)	
Liver	180			
Yes		19 (40.4%)	48 (36.1%)	0.603
No		28 (59.6%)	85 (63.9%)	
Lung and liver	180			
Yes		10 (21.3%)	28 (21.0%)	0.989
No		37 (78.7%)	105 (79.0%)	
CNS	180			
Yes		22 (46.8%)	57 (42.8%)	0.733
No		25 (53.2%)	76 (57.1%)	
Other visceral sites	180			
Yes		13 (27.7%)	40 (30.1%)	0.853
No		34 (72.3%)	93 (69.9%)	
Subsequent other treatment	171			
Yes		23 (48.9%)	70 (52.6%)	0.494
No		23 (48.9%)	55 (41.4%)	
Unknown		1 (2.1%)	8 (6.00%)	

BRAFi, BRAF inhibitor; CNS, central nervous system; CR, complete response; *N*, number of patients available for analysis; *n*, number of patients in the group; TBP, treatment beyond progression; ECOG, Eastern Cooperative Oncology Group; LDH, lactate dehydrogenase.

Of 171 evaluable patients with data on subsequent treatments, 59 (34.5%) did not receive any subsequent treatment after end of BRAFi treatment and 19 patients (11.1%) received only local treatment (radiation, surgery) of progressive sites. 93 patients (51.7%) received at least one further systemic treatment (Table 5), in 17 patients in combination with radiotherapy and in three patients with additional surgical interventions. Of the 93 patients with subsequent systemic treatment, 54 (58.1%) were treated with ipilimumab, 20 (21.5%) received a chemotherapy, 6 (6.5%) received targeted therapies (including other BRAFi, combination therapy of BRAFi/MEKi), 3 (3.2%) were treated with a PD‐1‐antibody, and 10 (10.8%) received other diverse therapies such as interleukin 2, RNA vaccines, or the multi‐kinase inhibitor lenvatinib. Again, patients with better prognostic features at progression such as ECOG 0 (*P *=* *0.0002), only new metastases (*P *=* *0.027) and single site progression (*P *=* *0.092) more frequently received further treatments (Table 5). 81.6% of patients did not respond at all to the subsequent treatment. Only two of three patients who received a PD‐1‐antibody and one patient treated with another BRAFi revealed an objective response. Notably, ipilimumab achieved no objective responses. One patient with BRAFi/MEKi (of 6; 16.7%), two patients with chemotherapy (of 19; 10.5%), and eight patients with ipilimumab (of 52; 15.4%) had a stable course of their disease. DC was achieved more often in patients with ECOG of 0 at progression compared to ECOG ≥1 (*P *=* *0.003).

**Table 5:** Subsequent systemic treatment after BRAFi progression

Risk factor	*N*	Patients with subsequent systemic treatment*n *=* *93 (51.7%)	Patients without subsequent systemic treatment*n *=* *78 (43.3%)	*P*‐value
Median age at progression (range), in years	171	52.3 (19.5; 86.3)	52.2 (26.1; 99.5)	0.612
BRAF mutation	171			
V600E		62 (66.7%)	56 (71.8%)	0.102
V600K		3 (3.20%)	7 (9.00%)	
Unknown		28 (30.1%)	15 (19.2%)	
Previous treatment (before BRAFi)	171			
Yes		46 (49.5%)	46 (59.0%)	0.215
No		47 (50.5%)	32 (41.0%)	
Elevated LDH at progression	132			
Yes		35 (37.6%)	31 (39.7%)	0.289
No		42 (45.2%)	24 (30.8%)	
Unknown		16 (17.2%)	23 (29.5%)	
Elevated S100 at progression	62			
Yes		22 (23.7%)	17 (21.8%)	0.419
No		16 (17.2%)	7 (9.00%)	
Unknown		55 (59.1%)	54 (69.2%)	
ECOG at progression	93			
0		23 (24.7%)	4 (5.10%)	
≥1		29 (31.2%)	37 (47.4%)	**0.0002**
Unknown		41 (44.1%)	37 (47.4%)	
Pattern of progression I	171			
New only		23 (24.7%)	12 (15.4%)	
Existing only		31 (33.3%)	17 (21.8%)	**0.027**
Both new and existing		39 (41.9%)	49 (62.8%)	
Pattern of progression II	171			
No CNS		49 (52.6%)	37 (47.4%)	0.128
CNS and other sites		22 (23.7%)	29 (37.2%)	
CNS only		22 (23.7%)	12 (15.4%)	
Pattern of progression III				
Preexisting in CR	168			0.469
Yes		12 (12.9%)	7 (9.00%)	
No		79 (84.9%)	70 (89.7%)	
Controlled despite progression	168			
Yes		72 (77.4%)	59 (75.6%)	0.992
No		20 (21.5%)	17 (21.8%)	
Single site progression				
Yes	171	19 (20.4%)	8 (10.3%)	
No		74 (79.6%)	70 (89.7%)	0.092
Sites of progression				
Lymph nodes metastases	171			
Yes		55 (59.1%)	49 (62.8%)	0.641
No		38 (40.9%)	29 (37.2%)	
Liver	171			
Yes		29 (31.2%)	34 (43.6%)	0.112
No		64 (68.8%)	44 (56.4%)	
Lung and liver	171			
Yes		15 (16.1%)	20 (25.6%)	0.133
No		78 (83.9%)	58 (74.4%)	
CNS	171			
Yes		38 (40.9%)	37 (47.4%)	0.440
No		55 (59.1%)	41 (52.6%)	
Other visceral sites	171			
Yes		27 (29.0%)	23 (29.5%)	0.990
No		66 (71.0%)	55 (70.5%)	

BRAFi, BRAF inhibitor; CNS, central nervous system; CR, complete response; *N*,‐number of patients available for analysis; *n*, number of patients in the group; TBP, treatment beyond progression; ECOG, Eastern Cooperative Oncology Group; LDH, lactate dehydrogenase.

### Prognostic factors at BRAFi progression for survival

**Figure 2:**
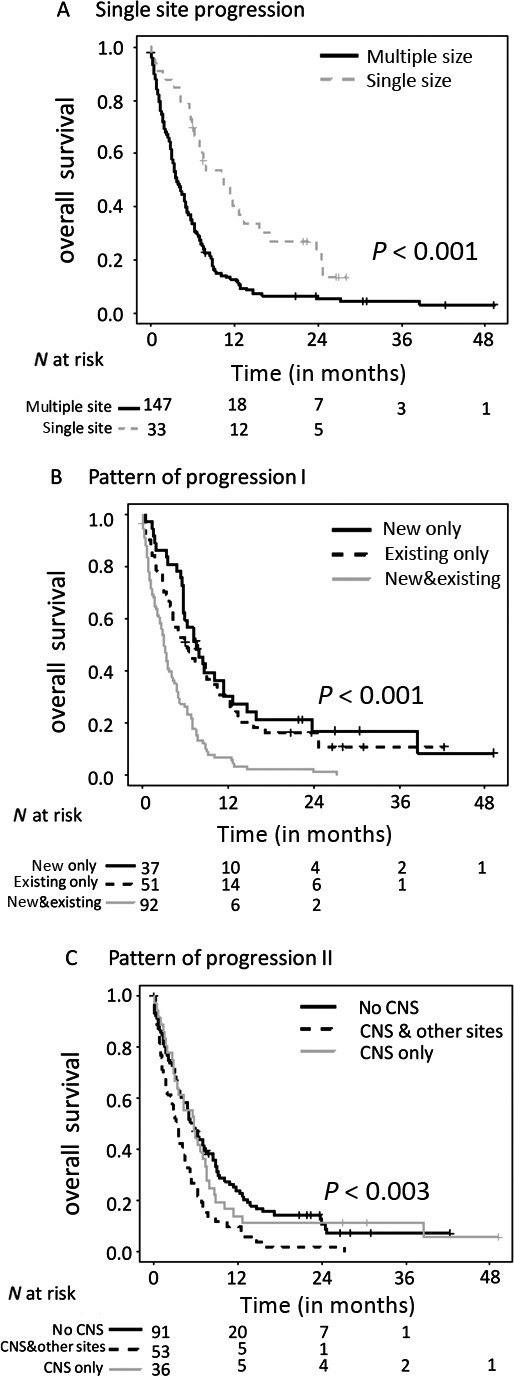
Overall survival after progression on BRAF inhibitors was significantly better in patients with (A) single site progression, (B) only progression of new *or* preexisting metastases (pattern of progression I), and (C) only CNS‐ *or* non‐CNS progression (pattern of progression II).

The median OS was significantly longer in patients with normal S‐100 at progression (11.4 months [95%CI 7.9–16.0, log‐rank *P *<* *0.001] vs. 3.5 months [95% CI 2.7–5.0] for the patients with elevated S‐100), with normal LDH at progression (6.6 months [95% CI 5.6–8.8, *P *=* *0.02] vs. 3.5 months [95% CI 2.3–5.3] for the patients with elevated LDH) and ECOG 0 at progression (10.9 months [95% CI 6.3–24.0, *P *<* *0.001] vs. 3.4 months [95% CI 2.7–5.3] for the patients with ECOG >1) (log‐rank test). Patients with progression with only new or only existing metastases (*P *<* *0.001), progression with only cerebral or only extracerebral metastases (*P *=* *0.001), and progression in only a single site (*P *<* *0.001) as well as patients without progression of bone metastases (*P *<* *0.001) had a better OS (Table S3, Figure 2). Patients with subsequent treatment after progression were more likely to have a longer OS. In contrast, TBP with the BRAFi had no significant impact on OS. Patients who received immunotherapy following BRAFi progression had a median OS of 6.7 months (95% CI 5.7–8.8) compared to 5.6 months (95% CI: 3.8–8.6) for patients with any other treatment such as targeted therapy, chemotherapy, and others (log‐rank test, *P *=* *0.155).

## Discussion

Progression of patients on treatment with a BRAFi can be fast and further treatments especially with immunotherapies such as ipilimumab had been of poor efficacy 13, 14. Hence, it is a matter of debate how to sequence treatments and if patients might benefit from BRAFi TBP. Here, we retrospectively analyzed a large cohort of patients who progressed on BRAFi therapy. Although we found an overall response rate of 52% prior to progression on BRAFi which is comparable to results of phase III trials 1, 2, 3, the median PFS and OS were only 4.6 and 9.8 months, respectively, and hence lower than in the phase III trials (median PFS between 5.1 and 8.8 months 2, 3, 4 and median OS between 15.9 and 18.7 months 3, 4). This difference can be explained by our patient selection criteria focusing on patients who eventually progressed on BRAFi with excluding the ones who achieved a long‐term tumor control by BRAFi 8, 15. This also explains why we have such a high number of primarily resistant patients in the cohort (23%). Additionally, more than half of our patients had received at least one prior therapy for metastatic disease compared to only treatment‐naïve patients 8 and no specification of pretreatment 15 in previous publications.

As formerly reported, progression on BRAFi is heterogeneous. The majority of our patients progressed in both new and preexisting metastases (52%) which is higher than reported previously in patients on BRAFi monotherapy (30%) and BRAFi + MEKi combination therapy (20%) 8, 15. Twenty‐one percentage of our patients progressed with only new metastases which is similar to data reported for BRAFi monotherapy (19%) 15, but different from a small phase I study on BRAFi therapy (42%) 9, and BRAFi + MEKi combination therapy (about 50%) 8, 16. We saw progression of preexisting metastases only in 28% of patients which is close to the 30% found by Long et al., whereas Chan et al. described that more frequently (48%) 8, 15. Possible explanations for these differences could be dissimilar patient characteristics (e.g., treatment‐naïve vs. pretreated), radiology review standards, and ultimately differences in BRAFi monotherapy versus BRAFi/MEKi combination. We found the longest PFS in patients with progression of only new *or* preexisting lesions, whereas Long et al. only found a better PFS in patients with progression of only preexisting lesions 8. The shortest OS in our patient cohort was seen in the group of patients with progression by new *and* preexisting metastases.

About half of the patients progressed in the CNS (49%) but only 20% without extracranial progression which is in line with published data 15. Nevertheless, in our study patients with only CNS or only extracranial progression did not show significant differences in OS. But, OS was significantly shortened in patients with progression of intra‐ *and* extracerebral metastases (30% of patients). This is interesting as Chan et al. surprisingly reported that the presence of brain metastases at time of progression was a factor for prolonged OS 15. However, Long et al. found a shorter OS for BRAFi/MEKi‐treated patients with new CNS metastases 8.

We found single site progression (18% of patients) significantly associated with prolonged OS. Others reported single site progression more frequently (31% of patients) 15 possibly explained by differences in patient selection; of note, our data were derived from 180 patients which is much larger than in the previous reports 15.

We could not find a significant impact of TBP on OS, neither on OS defined from start of BRAFi therapy nor on OS defined from time of first progression on the BRAFi which is in contrast to data of Chan et al. 15. Reasons for the significant impact of TBP on OS found by other authors might be multiple prognostic beneficial factors in their TBP group, for example, lower ECOG, normal LDH, single site progression, and absence of brain metastases 15. However, our TBP patients also showed more favorable prognostic factors than the non‐TBP patients including previous progression of only new or only existing metastases, a trend to normal LDH and BRAF V600E mutation. Nevertheless, we did not find a significant impact of TBP on OS. Hence, it is unlikely that TBP slows down the kinetics of progression under BRAFi treatment.

Concerning further systemic treatment after progression on the BRAFi we found a strikingly low activity of subsequent treatments. This may be explained by the time of treatment as our patient group has a long follow‐up and subsequent treatments that time were mainly ipilimumab and chemotherapy. Our data support previous reports on ipilimumab where no single patient benefited from the treatment after progression on a BRAFi 13. We can only draw limited conclusions on PD‐1 antibodies as only three of our patients received these. However, two of them showed an objective response and this is at least promising. Nevertheless, data from the Keynote‐006 trial show that PFS in BRAFi pretreated patients is shorter than in BRAFi‐naïve patients 17. Possible explanations include a cross‐resistance between BRAFi/MEKi and PD‐1 antibody resistance 18. Recent data indicate that a re‐challenge of BRAFi ± MEKi after progression on especially checkpoint inhibitors can be an efficacious treatment option 19, 20.

In summary, OS was especially limited in patients with an already impaired general condition (baseline ECOG ≥1), in patients who progressed with both, preexisting and new metastases, and at extra‐ and intracranial sites simultaneously. TBP did not prolong survival, other subsequent treatments such as ipilimumab and chemotherapy had a low efficacy with the exception of PD‐1 antibodies. Of course, conclusions from our study have to be drawn with care because of its retrospective character. Randomized trials for patients progressing on BRAFi ± MEKi are needed.

## Conflict of Interest Disclosures

J. C. Hassel received honoraria and travel reimbursements from Bristol‐Myers Squibb (BMS), Novartis, Merck Sharp and Dohme (MSD), Roche Pharma AG, Amgen, and Merck‐Serono. K. Buder‐Bakhaya received honoraria and travel reimbursements from TEVA GmbH, MSD, and Roche. C. Bender received honoraria and travel reimbursements from MSD and is (after her contribution to this work) an employee of Roche since 1 June 2016. L. Zimmer has served as consultant or/and has received honoraria from Roche, BMS, MSD, GlaxoSmithKline (GSK), Novartis, Merck, and travel support from MSD, BMS, Amgen and Novartis. B. Weide received grants and personal fees from BMS, MSD, personal fees from Roche, Curevac and Novartis, grants and personal fees from Philogen. C. Loquai received honoraria, travel reimbursements from BMS, Novartis, MSD, Roche, Amgen, Leo, Novartis, Pierre Fabre, and GSK. S. Ugurel declares advisory board and speakers honoraria from BMS, MSD and Roche, as well as grant and travel support from BMS, MSD, Roche und medac. R. Gutzmer received honoraria from Roche, BMS, GSK, Novartis, MSD, Merck‐Serono, Almirall, Amgen, Boehringer Ingelheim. He received travel reimbursements form BMS and Roche. He consults Roche, BMS, GSK, Novartis, MSD, LEO, Amgen, Pierre Fabre, and Pfizer and has received research funding from Pfizer and Johnson & Johnson. A. Slynko has no conflicts of interest.

## Supporting information


**Table S1.** Patients’ baseline characteristics and primary response to the BRAFi treatment.
**Table S2.** Patients’ characteristics at disease progression.
**Table S3.** Univariable response‐to‐therapy analyses.
**Table S4.** Univariable analyses for progression‐free survival (logistic regression).
**Table S5.** Univariable analyses for OS (Cox PH model).
