# Supplementary material for: Progression patterns under BRAF inhibitor treatment and treatment beyond progression in patients with metastatic melanoma
Source: Cancer Med. 2017 Dec 20;7(1):95–104. doi: 10.1002/cam4.1267 (PMC5773979; doi:10.1002/cam4.1267)
Supplement: Supplementary file 1 — Table S1. Patients’ baseline characteristics and primary response to the BRAFi treatment. Table S2. Patients’ characteristics at disease progression. Table S3. Univariable response‐to‐therapy analyses. Table S4. Univariable analyses for progression‐free survival (logistic regression). Table S5. Univariable analyses for OS (Cox PH model). [file CAM4-7-95-s001.docx]

**Suppl. Table 1: Patients’ baseline characteristics and primary response to the BRAFi treatment.**

| **Parameter** | **Total number of patients** | **Response groups**  **CR+PR SD PD** | | |
| --- | --- | --- | --- | --- |
| **Number of patients in each response group** | **180 (100%)** | **6+87 (51.7%)** | **45 (25.0%)** | **42 (23.3%)** |
| **Median age** [range], in years | **52 [19-99]** | **52** [19-99] | **51** [20-78] | **54** [26-77] |
| **Gender** |  |  |  |  |
| Male | **101 (56.0%)** | 50 (53.8%) | 23 (51.1%) | 28 (66.7%) |
| Female | **79 (44.0%)** | 43 (46.2%) | 22 (48.9%) | 14 (33.3%) |
| **ECOG-PS** |  |  |  |  |
| 0 | **50 (27.8%)** | 31 (33.3%) | 15 (33.4%) | 4 (9.5%) |
| ≥1 | **64 (35.6%)** | 32 (34.4%) | 10 (22.2%) | 22 (52.4%) |
| unknown | **66 (36.7%)** | 30 (32.3%) | 20 (44.4%) | 16 (38.1%) |
| **Number of previous therapies** |  |  |  |  |
| 0 | **82 (45.6%)** | 41 (44.1%) | 17 (37.8%) | 24 (57.2%) |
| 1 | **50 (27.8%)** | 29 (31.2%) | 12 (26.7%) | 9 (21.4%) |
| 2 | **29 (16.1%)** | 14 (15.1%) | 11 (24.4%) | 4 (9.5%) |
| ≥3 | **18 (10.0%)** | 9 (9.6%) | 5 (11.1%) | 4 (9.5%) |
| unknown | **1 (0.6%)** |  |  | 1 (2.4%) |
| **BRAF mutation type** |  |  |  |  |
| V600E | **123 (68.3%)** | 67 (72.0%) | 32 (71.1%) | 24 (57.1%) |
| V600K | **10 (5.6%)** | 1 (1.1%) | 4 (8.90%) | 5 (11.9%) |
| unknown | **47 (26.1%)** | 25 (26.9%) | 9 (20.0%) | 13 (31.0%) |
| **BRAFi** |  |  |  |  |
| Vemurafenib | **148 (82.4%)** | 76 (81.7%) | 33 (73.3%) | 39 (92.9%) |
| Dabrafenib | **32 (17.8%)** | 17 (18.3%) | 12 (26.7%) | 3 (7.1%) |
| **Treatment inside clinical trial/ EAP** |  |  |  |  |
| yes | **144 (80.0%)** | 77 (82.8%) | 39 (86.7%) | 28 (66.6%) |
| no | **35 (19.4%)** | 16 (17.2%) | 6 (13.3%) | 13 (31.0%) |
| unknown | **1 (0.6%)** |  |  | 1 (2.4%) |
| **Elevated serum LDH** |  |  |  |  |
| yes | **89 (49.4%)** | 35 (37.6%) | 27 (60.0%) | 27 (64.3%) |
| no | **73 (40.6%)** | 49 (52.7%) | 14 (31.1%) | 10 (23.8%) |
| unknown | **18 (10.0%)** | 9 (9.7%) | 4 (8.9%) | 5 (11.9%) |
| **Elevated S-100** |  |  |  |  |
| yes | **77 (42.8%)** | 39 (41.9%) | 22 (48.9%) | 16 (38.1%) |
| no | **26 (14.4%)** | 16 (17.2%) | 5 (11.1%) | 5 (11.9%) |
| unknown | **77 (42.8%)** | 38 (40.9%) | 18 (40.0%) | 21 (50.0%) |
| **Median time to best response**  (range), in months | **2.0 (0.4-13.9)** | 2.0 (0.82-13.0) | 1.8 (0.8-5.6) | 2.0 (0.4-13.9) |

**Suppl. Table 2: Patients’ characteristics at disease progression.**

| **Parameter** | **Total number of patients** | **Response groups**  **CR+PR SD PD** | | |
| --- | --- | --- | --- | --- |
| **ECOG-PS** |  |  |  |  |
| 0 | **30 (16.7%)** | 22 (23.6%) | 7 (15.6%) | 1 (2.40%) |
| ≥1 | **69 (38.3%)** | 33 (35.5%) | 15 (33.3%) | 21 (50.0%) |
| unknown | **81 (45.0%)** | 38 (40.9%) | 23 (51.1%) | 20 (47.6%) |
| **Elevated S-100** |  |  |  |  |
| yes | **42 (23.3%)** | 22 (23.6%) | 14 (31.1%) | 6 (14.3%) |
| no | **24 (13.3%)** | 17 (18.3%) | 4 (8.90%) | 3 (7.10%) |
| unknown | **114 (63.4%)** | 54 (58.1%) | 27 (60.0%) | 33 (78.6%) |
| **Elevated serum LDH** |  |  |  |  |
| yes | **68 (37.8%)** | 31 (33.3%) | 21 (46.6%) | 16 (38.1%) |
| no | **70 (38.9%)** | 47 (50.6%) | 12 (26.7%) | 11 (26.2%) |
| unknown | **42 (23.3%)** | 15 (16.1%) | 12 (26.7%) | 15 (35.7%) |
| **Pattern of progression I** |  |  |  |  |
| Only new metastases | **37 (20.6%)** | 24 (25.8%) | 5 (11.1%) | 8 (19.1%) |
| Only pre-existing metastases | **51 (28.3%)** | 32 (34.4%) | 12 (26.7%) | 7 (16.7%) |
| Both new and pre-existing metastases | **92 (51.1%)** | 37 (39.8%) | 28 (62.2%) | 27 (64.2%) |
| **Pattern of progression II** |  |  |  |  |
| Only CNS metastases | **36 (19.4%)** | 17 (18.3%) | 10 (22.3%) | 9 (21.4%) |
| Only extracerebral metastases | **91 (50.6%)** | 54 (58.1%) | 20 (44.4%) | 17 (40.5%) |
| CNS and extracerebral metastases | **53 (29.4%)** | 22 (23.6%) | 15 (33.3%) | 16 (38.1%) |
| **Pattern of progression III** |  |  |  |  |
| Progression of pre-existing metastases in CR | **19 (10.6%)** | 16 (17.2%) | 2 (4.4%) | 1 (2.4%) |
| Controlled metastases despite progression | **138 (76.7%)** | 72 (77.4%) | 38 (84.4%) | 28 (66.7%) |
| **Sites of progression ^*1^** |  |  |  |  |
| Lymph nodes | **107 (59.4%)** | 55 (59.1%) | 27 (60.0%) | 25 (59.5%) |
| Lung | **90 (50.0%)** | 45 (48.4%) | 20 (44.4%) | 25 (59.5%) |
| CNS | **79 (43.9%)** | 32 (34.4%) | 23 (51.1%) | 24 (57.1%) |
| Liver | **67 (37.2%)** | 27 (29.1%) | 19 (42.2%) | 21 (50.0%) |
| **Single site progression** |  |  |  |  |
| yes | **33 (18.3%)** | 22 (23.7%) | 8 (17.8%) | 3 (7.1%) |
| no | **147 (81.7%)** | 71 (76.3%) | 37 (82.2%) | 39 (92.9%) |
| **BRAFi treatment beyond progression (TBP)** |  |  |  |  |
| yes | **47 (26.1%)** | 24 (25.8%) | 8 (17.8%) | 15 (35.7%) |
| no | **133 (73.9%)** | 69 (74.2%) | 37 (82.2%) | 27 (64.3%) |
| **Subsequent systemic treatment** |  |  |  |  |
| yes | **93 (52.8%)** | 55 (59.1%) | 18 (40.0%) | 20 (47.6%) |
| no | **78 (43.3%)** | 32 (34.4%) | 26 (57.8%) | 20 (47.6%) |
| unknown | **9 (5.0%)** | 6 (6.5%) | 1 (2.20%) | 2 (4.80%) |
| **Type of subsequent systemic treatment** |  |  |  |  |
| Immunotherapy (CTLA-4 Ab or PD-1 Ab) | **57 (61.3%)** | 33 (35.5%) | 10 (22.2%) | 14 (33.3%) |
| Targeted therapy (BRAFi +/-MEKi)^*2^ | **6 (6.5%)** | 3 (3.20%) | 1 (2.20%) | 2 (4.8%) |
| Chemotherapy | **20 (21.5%)** | 10 (10.8%) | 7 (15.6%) | 3 (7.1%) |
| Other | **10 (10.8%)** |  |  |  |

**1: Most frequent sites of progression are listed.*

**2: This does not include TBP but patients who received BRAFi-reinduction therapy or who were switch to another BRAFi or sBRAFi/MEKi combination treatment.*

**Suppl. TABLE 3:** **Univariable response-to-therapy analyses.**

| **Risk factor** | **N** | **Response versus Non-Response groups** | | | **Disease Control versus Progression groups** | | |
| --- | --- | --- | --- | --- | --- | --- | --- |
|  |  | **OR** | **95%CI** | **p-value** | **OR** | **95%CI** | **p-value** |
| **Gender**  Male  Female | 180 | 1.22 | (0.67; 2.20) | 0.512 | 1.78 | (0.87; 3.75) | 0.118 |
| **Previous therapies**  yes  no | 180 | 1.16 | (0.64; 2.09) | 0.630 | 1.95 | (0.96; 4.0) | 0.065 |
| **BRAFi**  Vemurafenib  Dabrafenib | 180 | 1.07 | (0.5; 2.33) | 0.856 | 3.46 | (1.14; 15.0) | 0.051 |
| **Elevated LDH at baseline**  yes  no | 162 | **0.32** | **(0.16; 0.60)** | **< 0.001** | **0.36** | **(0.16; 0.79)** | **0.014** |
| **BRAF mutation**  V600E  V600K  unknown | 180 | **0.09**  0.95 | **(0.01; 0.52)**  (0.48; 1.87) | **0.026**  0.881 | **0.24**  0.63 | **(0.06; 0.93)**  (0.29; 1.41) | **0.035**  0.252 |
| **ECOG-PS at baseline**  0  $\geq$1 | 114 | 0.61 | (0.29; 1.29) | 0.202 | **0.17** | **(0.05; 0.48)** | **0.002** |
| **Single site progression**  yes  no | 180 | 2.14 | (0.99, 4.88) | 0.060 | **3.61** | **(1.2; 15.65)** | **0.043** |
| **Sites of progression**  *Liver*  yes  no  *Other visceral sites*  yes  no  *CNS*  yes  no    *Lung and liver*  yes  no  *Lymph nodes metastases*  yes  no | 180 | **0.48**  0.56  **0.45**  **0.40**  0.97 | **(0.26; 0.88)**  (0.29; 1.07)  **(0.24; 0.81)**  **(0.19; 0.84)**  (0.54; 1.77) | **0.020**  0.080  **0.009**  **0.017**  0.931 | 0.5  **0.3**  0.5  **0.36**  0.99 | (0.25; 1.0)  **(0.14; 0.62)**  (0.24; 1.0)  **(0.17; 0.79)**  (0.49; 2.0) | 0.053  **0.001**  0.050  **0.010**  0.990 |

**Suppl. TABLE 4:** **Univariable analyses for progression-free survival (logistic regression).**

| **Risk factor** | **N** | **OR** | **95%CI** | **p-value** |
| --- | --- | --- | --- | --- |
| **Elevated LDH at baseline**  yes  no | 162 | **0.37** | **(0.20; 0.71)** | **0.003** |
| **Elevated S100 at baseline**  yes  no | 103 | **0.37** | **(0.14; 0.93)** | **0.040** |
| **BRAF mutation**  V600E  V600K  unknown | 180 | 0.35  0.55 | (0.07; 1.31)  (0.27; 1.08) | 0.138  0.085 |
| **ECOG-PS at baseline**  0  ≥1 | 114 | **0.34** | **(0.16; 0.74)** | **0.007** |
| **Best response**  CR and PR  SD  PD | 180 | **0.27**  **0.03** | **(0.12; 0.56)**  **(0.01; 0.09)** | **< 0.001**  **< 0.001** |
| **Progress with lymph node metastases**  yes  no | 180 | **0.45** | **(0.24; 0.83)** | **0.010** |
| **Progress with bone metastases**  yes  no | 180 | **0.43** | **(0.20; 0.88)** | **0.023** |
| **Single site progression**  yes  no | 180 | **3.94** | **(1.73; 9.87)** | **0.002** |
| **Pattern of progression I**  new  existing  both new and existing | 180 | 1.25  **0.42** | (0.52; 3.00)  **(0.19; 0.91)** | 0.616  **0.029** |
| **Pattern of progression II**  no CNS  CNS and other sites  CNS only | 180 | 0.77  1.05 | (0.39; 1.53)  (0.48; 2.28) | 0.462  0.909 |
| **Pattern of progression III**  *pre-existing in CR*  yes  no  *controlled despite PD*  yes  no | 176  177 | 2.25  1.27 | (0.84; 6.68)  (0.62; 2.62) | 0.118  0.507 |

**Suppl. TABLE 5: Univariable analyses for OS (Cox PH model).**

| **Risk factor** | **N** | **Median OS (95%CI),**  **in months** | **HR** | **95% CI** | **p-value** |
| --- | --- | --- | --- | --- | --- |
| **OS I (beginning at BRAFi start)** | | | | | |
| **ECOG-PS at baseline**  0  ≥1 | 114 | 15.13 (11.6;20.5)  8.27 ( 7.0; 9.83) | **2.59** | **(1.72; 3.90)** | **< 0.001** |
| **BRAF mutation**  V600E  V600K  unknown | 180 | 10.62 (9.3; 13.4)  8.51 (4.9; 15.4)  9.06 (7.1; 11.0) | 1.55  1.14 | (0.81; 2.96)  (0.80; 1.62) | 0.189  0.478 |
| **OS II (beginning at BRAF progression)** | | | | | |
| **Pattern of progression I**  new only  existing only  both new & existing | 180 | 7.58 (5.80; 12.58)  6.29 (4.27; 10.52)  3.10 (2.53; 4.14) | 1.22  **2.77** | (0.77; 1.94)  **(1.82; 4.23)** | 0.401  **< 0.001** |
| **Pattern of progression II**  no CNS  CNS and other sites  CNS only | 180 | 5.66 (4.37; 7.78)  3.29 (2.00; 4.90)  5.75 (3.32; 7.48) | **1.80**  1.09 | **(1.26; 2.57)**  (0.72; 1.63) | **0.001**  0.689 |
| **Pattern of progression III**  *pre-existing in complete remission*  yes  no  *controlled despite progression*  yes  no | 176  177 | 6.29 (4.18; 24.5)  4.90 (3.52; 5.80)  5.13 (4.18; 6.24)  3.52 (1.87; 7.78) | 0.62  0.87 | (0.37; 1.04)  (0.60; 1.26) | 0.067  0.468 |
| **Progress with bone metastases**  yes  no | 180 | 2.65 (1.38; 4.77)  5.38 (4.28; 6.93) | **1.95** | **(1.35; 2.82)** | **< 0.001** |
| **Single site progression**  yes  no | 180 | 10.52 (6.9; 17.26)  3.78 (3.18; 4.97) | **0.45** | **(0.30; 0.69)** | **< 0.001** |
| **TBP**  yes  no | 180 | 5.98 (4.34; 7.45)  4.24 (3.29; 5.23) | 0.82 | (0.58; 1.17) | 0.279 |
| **Subsequent treatment**  yes  no | 171 | 6.71 (5.80; 8.76)  2.00 (1.35; 3.10) | 0.35 | (0.22; 0.55) | **<0.001** |

***Abbreviations****: BRAFi –BRAF inhibitor, CNS – central nervous system, CR -complete response, CTLA-Ab – CTLA4-antibody, ECOG-PS – Eastern Cooperative Oncology Group Performance Score, HR –hazard ratio, , LDH – lactate dehydrogenase, MEKi – MEK inhibitor, N -number of patients available for analysis, OR - odds ratio, OS – overall survival, PD - progressive disease, PD-1 Ab – PD-1-antibody, PR - partial response, SD - stable disease, TBP – treatment beyond progression.*
